# Development of a big data platform for collecting and utilizing clinical information from the Korea Biobank Network

**DOI:** 10.1186/s12911-025-03192-4

**Published:** 2025-10-08

**Authors:** Yun Seon Im, Seol Whan Oh, Ki Hoon Kim, Wona Choi, In Young Choi

**Affiliations:** 1 Department of Medical Sciences, College of Medicine, The Catholic University of Korea, Seoul, Republic of Korea; 2 Department of Medical Informatics, College of Medicine, The Catholic University of Korea, 222 Banpo-daero, Seocho-gu, Seoul, 06591 Republic of Korea

**Keywords:** Biobank, Biorepository, Electronic health record, Data processing, Information management, Database management systems, Survey

## Abstract

**Background:**

Advanced biobanks increasingly focus on supporting biomedical research through the collection and integration of large-scale biological and clinical datasets. This study aimed to develop a big data platform that enables institutions within the Korea Biobank Network (KBN) to efficiently collect and utilize clinical information using a standardized common data model.

**Methods:**

The KBN Biobank Research Information and Digital Image Exchange (BRIDGE) platform was developed to allow 43 biobanks to systemically collect and upload electronic medical records and clinical data. This platform was designed to incorporate automated quality verification and basic statistical preprocessing functionalities, allowing users to analyze data efficiently without complex queries. Additionally, a survey was conducted to evaluate user satisfaction with the platform.

**Results:**

Through the KBN BRIDGE platform, institutions collected and integrated clinical information on 39 diseases. A total of 136,473 patients’ clinical data, collected by institutions between 2021 and 2023, were uploaded to the KBN common data model, including 43,330 serum samples, 33,352 plasma samples, and 22,279 buffy coat samples. A satisfaction survey conducted among 35 institutional data managers reported an average score of 3.5 out of 5 for the platform.

**Conclusions:**

This study developed and demonstrated that the KBN BRIDGE platform enables institutions to systematically collect, integrate, and manage large-scale clinical information across multiple biobanks. Furthermore, through data quality management and preprocessing statistical functions, the platform has shown potential for several research applications. Future improvements in system functionality and clinical information utilization can further enhance the platform’s utility across various research fields.

**Supplementary Information:**

The online version contains supplementary material available at 10.1186/s12911-025-03192-4.

## Introduction

The use of modern biobanks increasingly highlights the importance of large-scale biological resources and clinical information in advancing medical research [1, 2]. Biobanks vary by research purpose, scale, and operational model, and can be based, for example, on single medical institutions, healthcare systems, or population cohorts [3]. Particularly, the integration of biobanking with electronic health records enriches biomedical research by providing detailed information on disease phenotypes; this was previously unavailable [4, 5]. Furthermore, electronic health records contain diverse clinical information, including patients’ medical histories, diagnoses, tests, treatments, and procedures. However, the structural and format differences in medical systems across institutions necessitate a systematic platform for data integration and accessibility [6].

International biobanks continually seek to enhance the collection and utilization of diverse data types [7, 8]; this reflects the growing need for large datasets that can effectively leverage the ever-expanding biobank resources and support the understanding of complex disease mechanisms over time [9]. Researchers use these extensive resources to develop new treatments and conduct studies on disease prevention and therapy. For example, in the United Kingdom, the UK Biobank has collected vast genetic and clinical information from over 500,000 participants, facilitating easy access for researchers and contributing to the development of disease prediction models and personalized therapies [10, 11]. In Japan, the biobank has gathered clinical information on cancer and chronic diseases from over 200,000 participants, supporting targeted research on these conditions [12]. The All of Us project in the United States collects data from diverse populations to advance personalized medicine by considering factors, such as electronic health records data, physical measures, and whole-genome sequences data [13, 14]. These pioneering efforts in biobanking are driving progress in disease research and treatment by integrating clinical information derived from population-based studies. To support such initiatives, several international data models have been developed. The Observational Medical Outcomes Partnership (OMOP) is widely utilized for observational studies by integrating EHR and claims data [15, 16]. The Informatics for Integrating Biology and the Bedside (i2b2) is software designed for clinical data warehousing that supports federated queries across multiple hospitals [17]. The Minimal Information About Biobank Data Sharing (MIABIS) serves as a minimal information standard designed primarily for metadata sharing and summary statistics at the biobank level [18]. These models aim to standardize the structure and semantics of biobank and clinical data, thereby facilitating data sharing, integration, and interoperability across institutions and countries.

The Korea Biobank Project was launched in 2008 at a national level to collect and manage bioresources in South Korea [19]. Through this project, the Korea Biobank Network (KBN) was established, which focuses on collecting clinical and genetic information on diseases that frequently affect Koreans or are specific to Korean health patterns. From 2019 to 2021, the KBN bioresources sharing and open platform initiative refined the management system for clinical and epidemiological data. This initiative has enabled Korean biobanks to gather a wide array of clinical information beyond specific diseases. As previously reported by Ko et al. (2021), an initial version of the KBN CDM was developed, encompassing 27 disease domains across 17 participating biobanks [20]. This preliminary effort established a basic data model structure supported by manual data collection through Excel spreadsheets, introduced standardized definitions for common clinical variables, and demonstrated a proof-of-concept framework for data integration. Despite its foundational role, the previous system relied on manual data entry, which was susceptible to human error and constrained the model’s scalability. In addition, data quality assurance was difficult to maintain due to the retrospective nature of data management. These constraints impeded the capacity to maintain consistent and real-time updates across a broad biobank network. Therefore, further improvements are needed to facilitate the integration of large-scale clinical data and enhance the utilization of high-quality KBN resources for users.

Consequently, a platform that can support the efficient collection and utilization of extensive clinical information is necessary. Therefore, this study aimed to develop a biobank big data platform, the Korea Biobank Network (KBN) Biobank Research Information and Digital Image Exchange (BRIDGE), to support the systematic collection and management of clinical information on various diseases by institutions within the KBN. Moreover, this platform aimed to provide an environment that enables institutions within the KBN to integrate their clinical data and includes data quality management functions to support its effective use.

## Methods

### The KBN BRIDGE system architecture

**Fig. 1:**
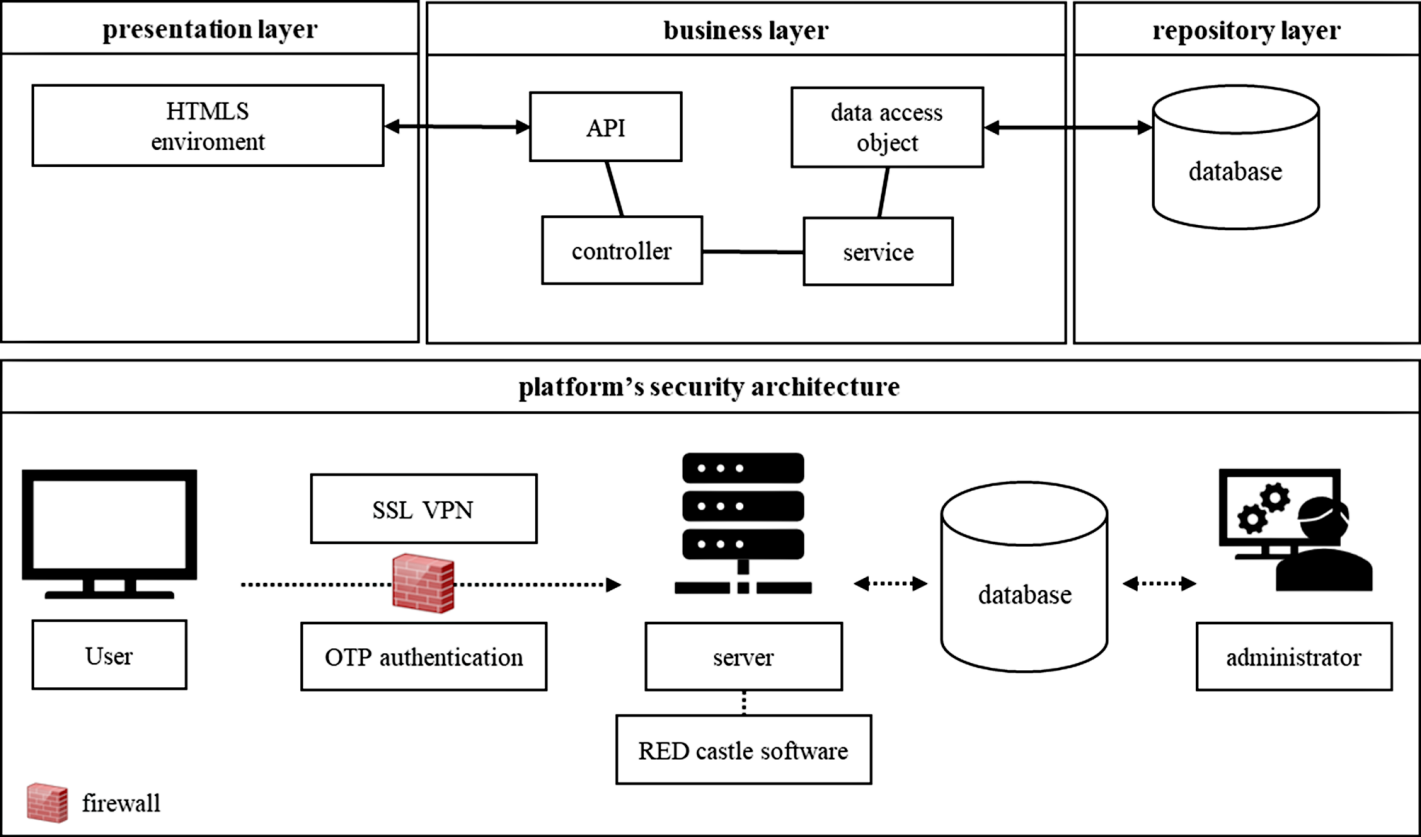
Korea Biobank Network (KBN) Biobank Research Information and Digital Image Exchange (BRIDGE) system’s architecture

The KBN BRIDGE platform was developed using Spring Framework (Tanzu Spring, Palo Alto, CA, USA; https://spring.io/projects/spring-framework) (Fig. 1). The presentation layer is compatible with hypertext markup language 5 (HTML5)-based web browsers, allowing data interaction between users and the system via application programming interfaces (APIs), where business logic is processed. The controllers and services primarily execute business logic in business layer, and batch processes generate the necessary statistical data for each interface. The repository layer generates data for each screen, managing the database and storage. The MySQL management system (Oracle Corp., Santa Clara, CA, USA; https://github.com/mysql/mysql-server) is used for the database operations.

The Platform‘s security solution combines multiple technologies to ensure the safe handling of medical data (Fig. 1). This includes applying secure sockets layer (SSL) encryption to data transmitted within the system; the use of a virtual private network (VPN) limits access to the internal network, blocking external intrusions system security is monitored in real-time using RED Castle 4.0 software, (SGA Solutions Co., Ltd., Uiwang, Republic of Korea), which provides automated responses to potential threats. To enhance security, the system employs one-time password (OTP) authentication, generating a unique password each time a user accesses the platform [21]. System administrators manage user account access permissions, maintain activity logs, and regularly inspect the system’s security status.

### The KBN common data model

The platform integrates detailed clinical information associated with biobank resources. Clinical data collected from various institutions and sources often differ in structure, format, and terminology, which complicates multi-institutional collaborative research [22]. We acknowledged the importance of interoperability and conducted a systematic evaluation of existing international common data models. The OMOP does not inherently support biospecimen tracking or provide the granular linkage between biospecimen samples and clinical data that is essential for biobank research [15, 16]. The i2b2 does not standardize cross-institutional data collection protocols for varying EHR system [17]. MIABIS 2012 does not encompass patient-specific clinical information or detailed specimen collection data, and therefore lacks the capability to link biospecimen information at an individual level [18]. MIABIS 2020 extensions introduced Sample Donor, Sample, and Event components that provide minimal clinical attributes such as diagnosis, sample collection timestamps, and donor demographics, while remaining primarily focused on biospecimen metadata [23]. A detailed comparison of existing data model and the KBN common data model is presented in Additional file 1: Table S1.

Existing data models did not provide a practical framework capable of harmonizing these diverse data sources while also meeting the requirements of Korean biobanks. The KBN comprised 43 biobanks utilizing heterogenous EHR systems in 2021. To address this issue, we developed the KBN Common Data Model (CDM) to enable the seamless integration of data from multiple institutions. The KBN CDM was specifically tailored for the Korea Biobank Network (KBN) to standardize and integrate clinical information. It encompasses 38 diseases and includes data from 43 biobanks, with information on non-disease participants.

The development of the KBN CDM was a collaborative effort by the National Institute of Health, Korea Disease Control and Prevention Agency, the Department of Medical Informatics, College of Medicine, The Catholic University of Korea, and other KBN institutions. A working group comprising principal investigators, data managers, and database developers from these establishments defined data collection items and constructed a standardized data model for the KBN CDM. While maintaining core concepts prevously reported by Ko et al. (2021), we enhanced the CDM structure by redesigning 70% of data elements, adding new tables for complex specimen-clinical linkages [20]. This model comprises 15 tables, 152 variables, and 1,852 codes (Table 1).

The KBN CDM is designed to standardize data extraction, transformation, and loading processes within KBN healthcare institutions, covering patients who are KBN donors, registration, specimens, body measurements, drinking and smoking, disease history, drugs, family history, laboratory examinations, notes, note details, disease-specific information, treatments, symptoms, follow-up data. The codes for these data are managed within a code table, including codes for disease diagnoses, body measurements, disease history, drug units, family history, laboratory examinations, note details, disease-specific information, treatments, and symptoms. Standardized terminology mapping is performed for codes that can be clearly matched, using internationally recognized vocabularies such as Systematized Nomenclature of Medicine Clinical Terms (SNOMED CT), Logical Observation Identifiers Names and Codes (LOINC), and Anatomical therapeutic chemical classification system (ATC) [24–26].

**Table 1:** Korea biobank network (KBN) common data model (CDM) data table information

Table	Columns	Description
PERSON	6	KBN donor identification, biobank name, sex, birth date, job, job detail
REGISTRATION	13	Disease diagnosis date, disease diagnosis code, disease characterization status, Korean Standard Classification of Diseases (KCD) version, disease diagnosis Korea name, disease diagnosis English name, disease diagnosis
SPECIMEN	7	Specimen identification, KBN donor identification, registration identification, specimen date, specimen code, specimen detail, specimen sum
BDMEASURE	6	Body measurement identification, KBN donor identification, registration identification, body measurement date, body measurement code, body measurement result
DRINSMOK	22	Drinking & smoking identification, KBN donor identification, registration identification, drinking & smoking record date, drinking experience, drinking duration, drinking unit, quit drinking duration, quit drinking unit, drinking frequency, drinking frequency unit, drinking amount, drinking amount unit, drinking type, etc., smoking experience, smoking duration, smoking unit, quit smoking duration, quit smoking unit, smoking amount, smoking pack year
HISTORY	8	Disease history identification, KBN donor identification, registration identification, date of first diagnosis, disease history record date, disease history type, disease history name, disease history, etc.
DRUG	15	Drug identification, KBN donor identification, registration identification, drug reference, drug name, National Health Insurance Service Electronic Data Interchange (EDI), Anatomical Therapeutic Chemical Classification (ATC), Prescription Normalized Naming System (RxNorm), drug dose, drug dose unit, drug dose, etc., drug dose times, drug start date, drug end date
FAMHISTORY	7	Family history of the disease identification, KBN donor identification, registration identification, family history of the disease record date, family history relations, etc., family history, etc.
CLINEXAM	10	Laboratory examination identification, KBN donor identification, registration identification, laboratory examination date, laboratory examination detail, laboratory examination result of numeric type, laboratory examination result of character for numeric, laboratory examination unit, laboratory examination result of character type, laboratory examination reference value
NOTE	7	Note identification, KBN donor identification, registration identification, note date, note type, note name, note text
NOTE_DETAIL	7	Note detail identification, KBN donor identification, registration identification, note identification, note detail date, note detail type, note detail result
DISEASE_SPECIFIC	6	Disease-specific identification, KBN donor identification, registration identification, disease-specific record date, disease-specific type, disease-specific result
TREATMENT	6	Treatment identification, KBN donor identification, registration identification, treatment date, treatment name, treatment detail, treatment result
SYMPTOM	7	Symptom identification, KBN donor identification, registration identification, symptom record date, symptom name, symptom result, symptom date
FOLLOWUP	26	Follow-up identification, KBN donor identification, registration identification, follow-up record date, death y/n, death date, cause of death code, KCD version, cause of death_1, cause of death_2, cause of death_3, cause of death_4, cause of death, etc., recurrence y/n, recurrence date, recurrence site, metastasis y/n, metastasis date, metastasis site, lung metastasis, lung metastasis diagnosis date, lung cancer, International Classification of Diseases for Oncology (ICD_O), TNM Classification of Malignant Tumors stage, therapy

**Fig. 2:**
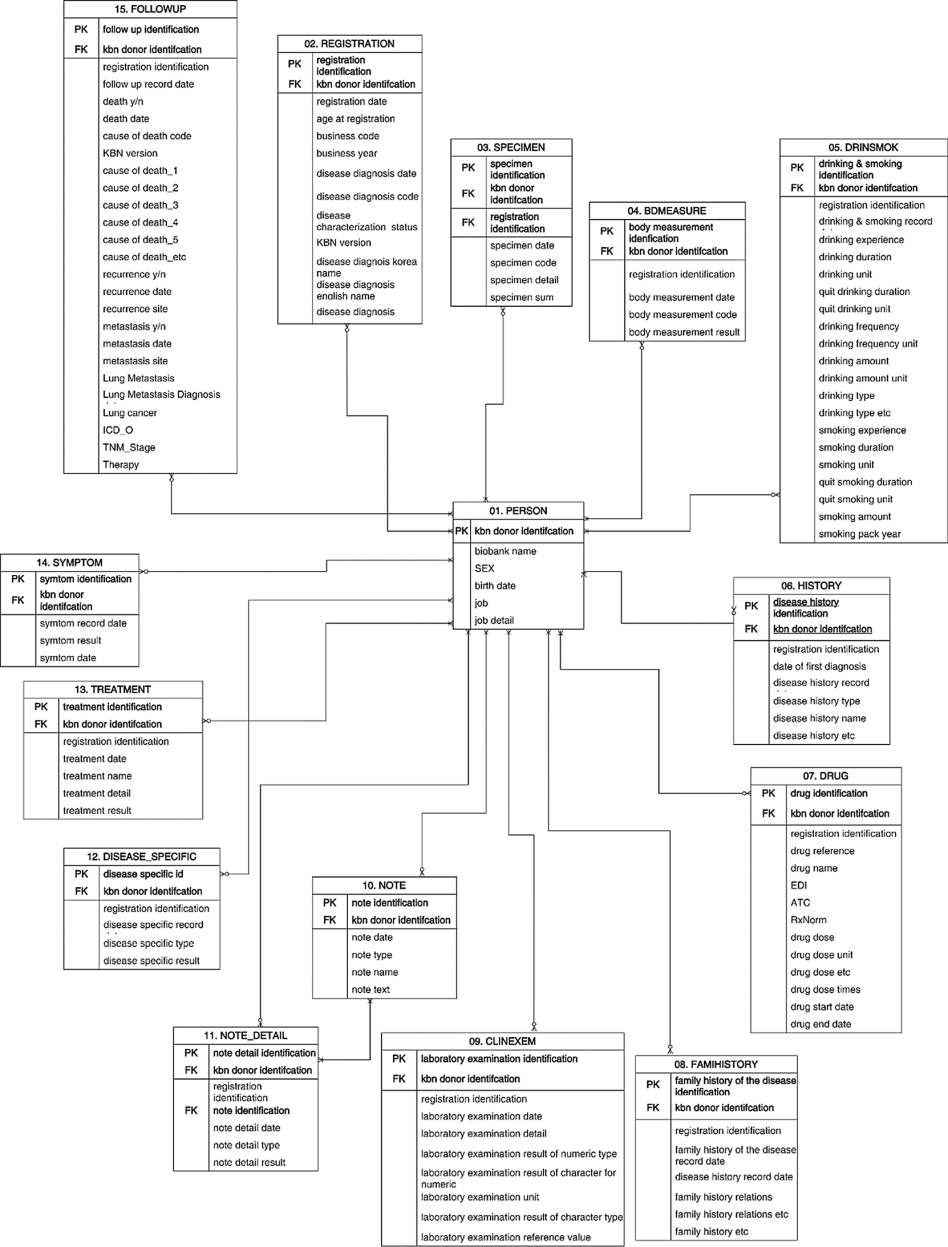
Korea Biobank Network (KBN) common data model (CDM)’s unified modeling language

The PERSON table collects basic demographic information on patients from each hospital and is central to the relationships with the other tables within the KBN CDM. This table maintains a one-to-many (1:N) relationship with other tables, where the unique patient identifier, KBN_ID, is composed of an institution code and a serial number (Fig. 2). In this process, an anonymized barcode provided by the Human Biobank Information System for Sample (HuBIS_Sam), a human biospecimen management program used by the Korea National Biobank to collect, store, manage quality, and distribute biospecimens, is utilized to ensure data security, containing no identifiable information. Furthermore, the REGISTRATION table stores data on the year of specimen collection and diagnostic codes for 38 diseases at the time of specimen intake, while the SPECIMEN table manages detailed specimen information linked to the HuBIS_Sam system used by the KBN, associating specimen codes and detailed specimen codes provided by HuBIS_Sam [27]. In response to specific requests from lung cancer researchers, the FOLLOWUP table now includes additional columns for information on lung cancer metastasis. For other diseases, the newly updated items are automatically integrated and tracked in the FOLLOWUP table. This design demonstrates the flexibility of the KBN CDM in accommodating disease-specific characteristics and evolving research needs.

### Electronic medical record or clinical data warehouse data linkage

**Fig. 3:**
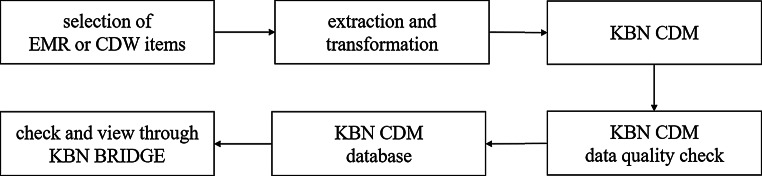
Korea Biobank Network (KBN) Biobank Research Information and Digital Image Exchange (BRIDGE) platform’s data process

The KBN organizes the 43 biobanks into 12 consortia, each comprising a primary institution and multiple collaborating institutions. The primary institution collects data from the collaborating institutions and uploads it to the KBN BRIDGE platform (Fig. 3). These biobanks perform extract, transform, and load (ETL) processes to convert electronic medical record (EMR) or clinical data warehouse (CDW) data from their respective medical institutions into the KBN CDM format. Data collection methods include comma-separated value (CSV) file uploads and application programming interface (API) transmission, with API transmission being preferred for its efficiency in handling large datasets [28].

However, implementing a unified ETL process provided challenging due to the heterogeneous IT environments across the 43 participating hospitals. Each institution operates distinct systems with unique database schemas, data formats, and access protocols. The spectrum included large university hospitals with dedicated IT teams as well as smaller hospitals with limited resources. Furthermore, due to data security and privacy regulations, direct access to institutional EMRs was prohibited.

To address these challenges, we provided each institution with a comprehensive ETL guideline that specifies target data formats for the KBN CDM, transformation rules, validation criteria, and standard code mappings. Additionally, we included sample scripts and templates based on a pilot project (with identifying details removed) that served as a practical reference to encourage broader participation. Each consortium received government funding to develop its own ETL infrastructure, allowing them to hire technical personnel or outsource development based on their specific EMR systems.

The collaborating institutions develop customized ETL scripts in accordance with the guideline, extract and transform their EMR data into the KBN CDM format, and perform initial quality checks. The primary institutions then aggregate the data, conduct consortium-level quality validation, and upload the consolidated dataset to the KBN BRIDGE platform. Users can access, manage, and download the integrated data directly through the KBN BRIDGE platform, enabling efficient management and utilization of clinical information collected by system users. Throughout this process, our team provided ongoing technical consultation, troubleshooting support, and regular training sessions.

### Data quality check of the KBN BRIDGE platform

**Fig. 4:**
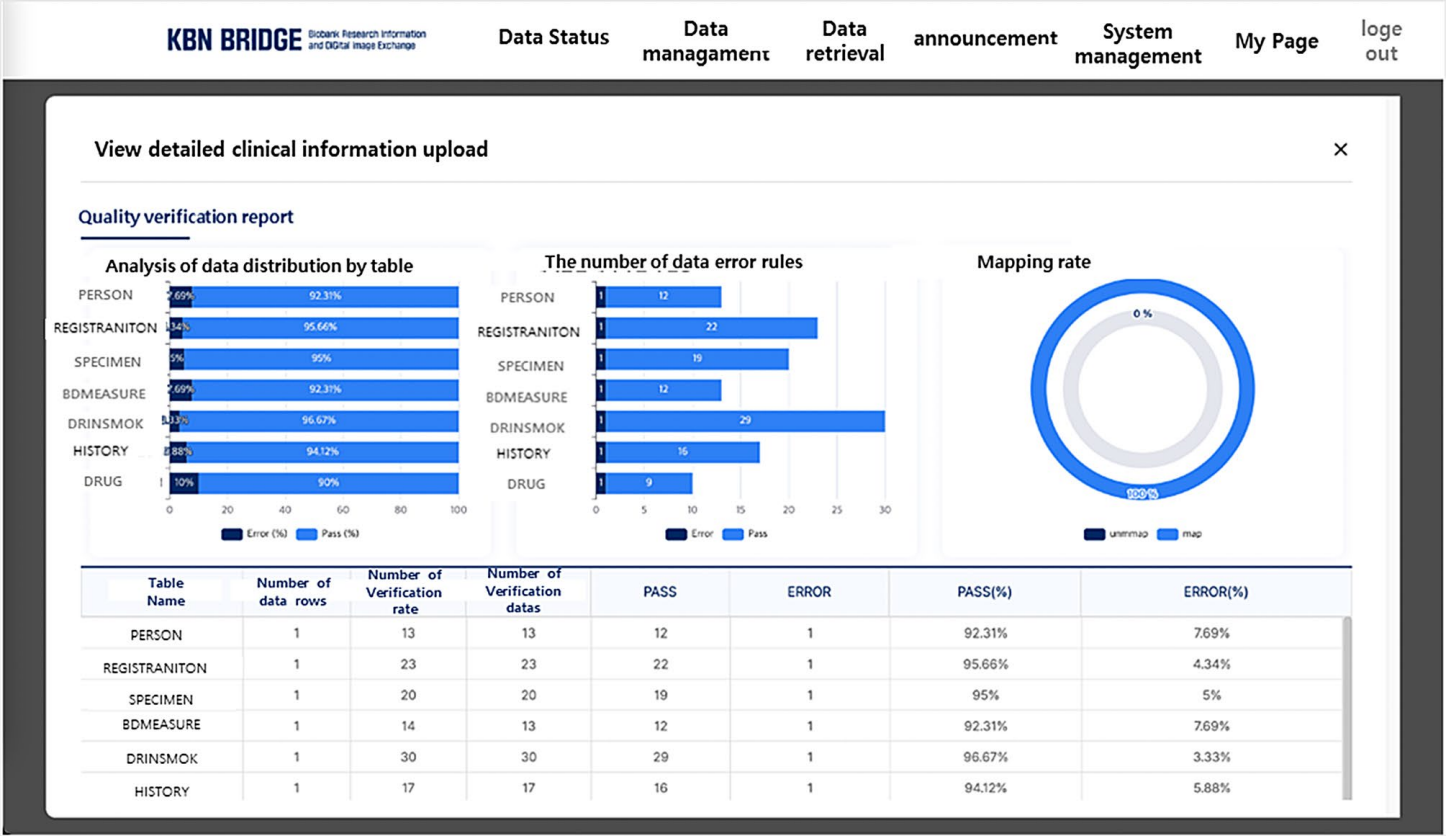
Korea Biobank Network (KBN) platform’s data quality report screenshot

Maintaining consistent quality in the clinical and epidemiological data collected from multiple institutions is essential, as differences in data structure and format can complicate integration and analysis [29]. To ensure data quality, the KBN BRIDGE platform includes a data quality management system. This system provides users with a quality validation report, allowing them to verify how their data is being validated. Furthermore, only data that passes all quality rules is ultimately loaded into the KBN CDM database, while data with errors is automatically rejected [30]. The quality validation report (Fig. 4) comprehensively visualizes the quality status of the entire dataset through charts and tables, displaying errors by table and quality rule type, along with validation results for error-free data. In addition, users can save these reports as CSV files, enabling them to quickly identify and correct erroneous data.

### Basic statistical preprocessing of the KBN BRIDGE platform

**Fig. 5:**
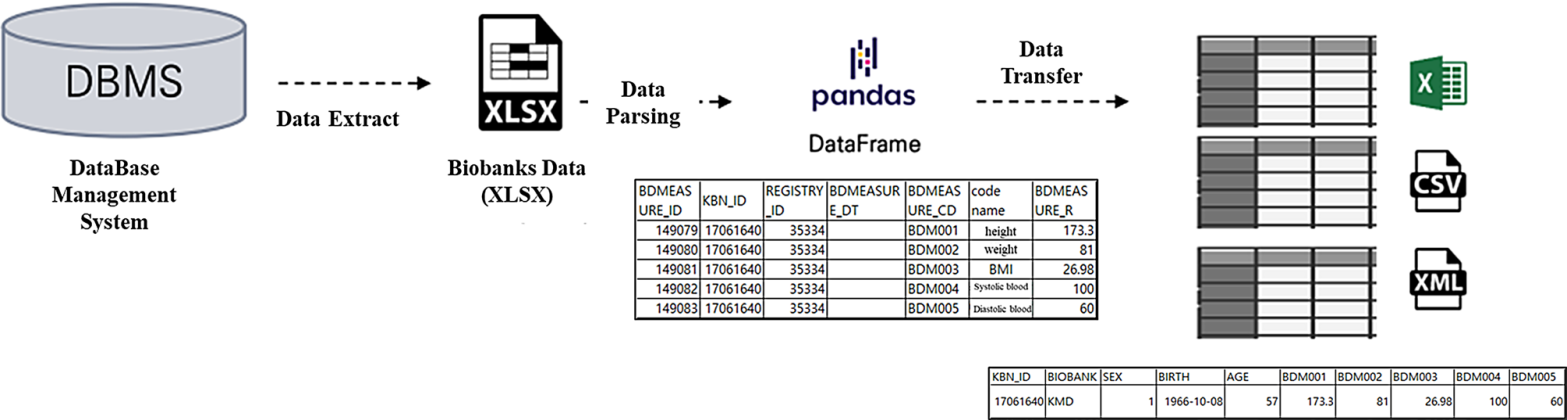
Korea Biobank Network (KBN) Biobank Research Information and Digital Image Exchange (BRIDGE) platform’s basic statistical preprocessing process

To facilitate user access to collected data, the KBN BRIDGE platform provides basic statistical preprocessing functions (Fig. 5). The biobank structures the collected dataset within a database; for users less familiar with database structures, it provides preprocessing tables and source code for the five primary tables (body measurements, drinking and smoking, disease history, family history, and laboratory examinations). This preprocessing process, implemented using Python pandas (NumFOCUS Inc., Austin, TX, USA. https://github.com/pandas-dev/pandas), includes combining patient information to check and remove missing and outlier values. Additionally, matrix transformations and basic statistical analyses are performed automatically, allowing users to process data quickly and easily without complex queries. The data is provided based on the patient’s most recent results. Therefore, the preprocessed files enable users to comprehensively view clinical information for each patient, supporting a more intuitive and efficient approach to data analysis.

### Satisfaction evaluation of the KBN BRIDGE platform

A survey was conducted with 35 biobank institutional data managers to evaluate user satisfaction with the KBN BRIDGE system. The satisfaction survey items were adapted to suit the objectives of this study [31, 32]. The survey employed a 5-point Likert scale, allowing respondents to rate each item from *strongly disagree* (1) to *strongly agree* (5), and included five items designed to measure overall user satisfaction with the system. Additionally, respondent profiles, such as gender, age group, career experience, and basic hospital information, were collected. Descriptive statistical methods were used to analyze the data.

## Results

### Clinical information collected through the KBN BRIDGE platform

Between 2021 and 2023, institutions within the KBN collected clinical data from a total of 136,473 patients and uploaded it to the KBN BRIDGE platform. Among them, 66,913 (49%) were male, and 69,560 (51%) were female. The patients’ age distribution varied widely, with the majority in their 50s (*n* = 31,582, 18.14%) and 60s (*n* = 42,736, 24.54%) (Table 2).

**Table 2:** Korea biobank network (KBN) biobank research information and digital image exchange (BRIDGE) platform’s dataset: patient basic demographics data (*n* = 136,473)

Variables	Category	Total *N* (%)
Sex	Male Female	66,913 (49) 69,560 (51)
Age (years)	Less than 10 10–19 20–29 30–39 40–49 50–59 60–69 70 years or older	5194 (3.8) 5820 (4.3) 10,608 (7.8) 18,087 (13.3) 22,706 (16.6) 31,582 (23.1) 42,736 (31.3) 37,350 (27.4)
Total		136,473

**Fig. 6:**
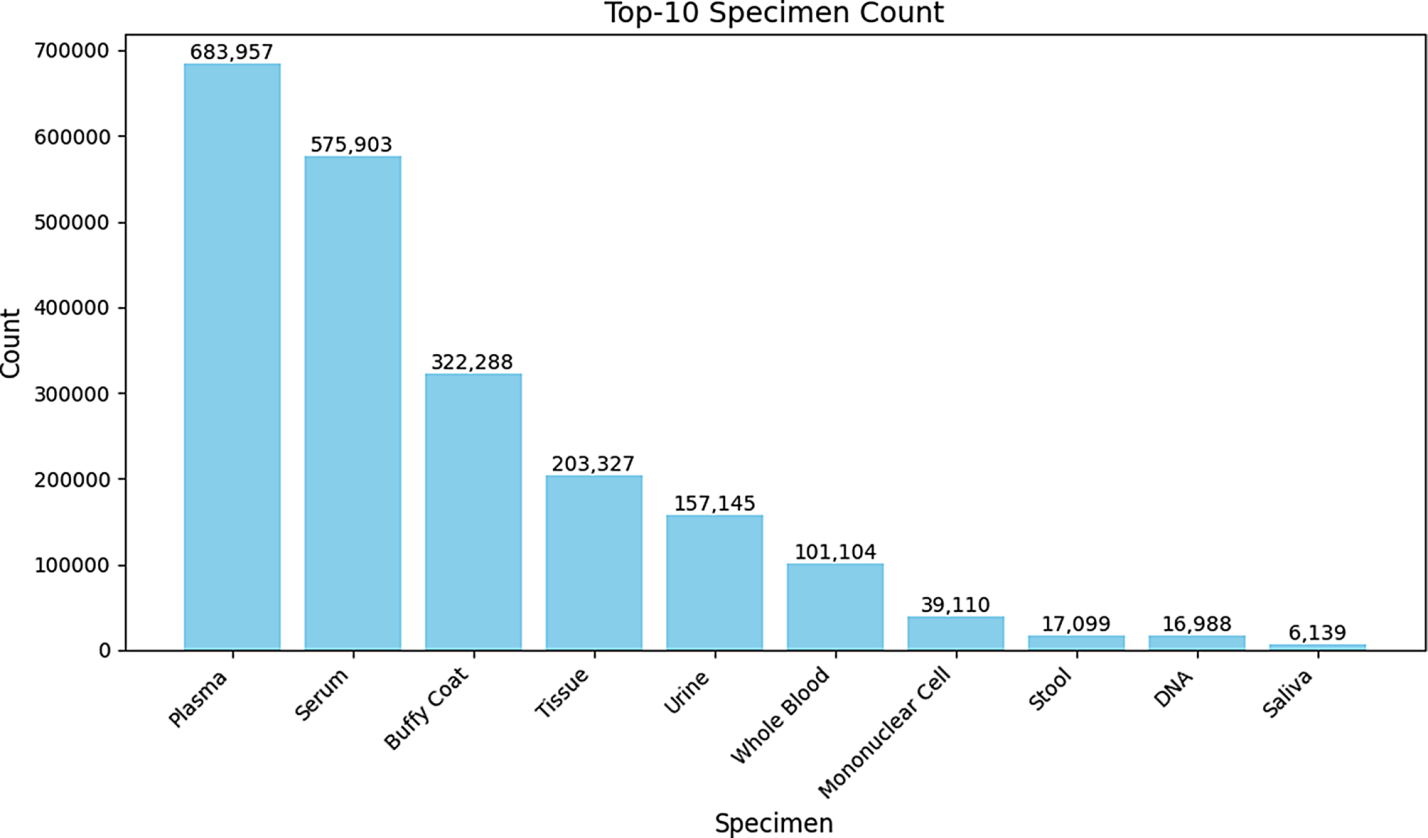
Korea Biobank Network (KBN) Biobank Research Information and Digital Image Exchange (BRIDGE) platform’s top 10 specimen categories

Figure 6 shows the top 10 specimen categories collected by KBN institutions through the KBN BRIDGE platform between 2021 and 2023. A total of 1,396,778 specimens were collected, with serum representing the largest proportion (433,303 specimens, 31.02%). Plasma (333,528 specimens, 23.87%) and buffy coat (222,786 specimens, 15.94%) were also among the most collected specimens. These three blood-related specimens accounted for over 70% of the total sample count. These top three specimen types (serum, plasma, and buffy coat) are critical for biomedical research, particularly in studies investigating cancer, immune responses, and gene expression [33, 34].

Furthermore, the 39 diseases were categorized into cancer-related diseases, non-cancer diseases, non-disease participants, and other diseases (not categorized in the 38 diseases Groups). Excluding non-disease participants and other diseases, blood cancers (*n* = 10,920, 6.27%) were the most frequently recorded. The least common condition was preterm birth (Table 3).

**Table 3:** The platform’s disease count categories

Variable	Category	Total *N* (%)
Disease	Hematologic malignancies Oral diseases Breast cancer Colorectal cancer Thyroid cancer Prostate cancer Lung cancer Liver cancer Glomerular disease, renal Kidney transplantation Bladder cancer Gastric cancer Female reproductive Gynecologic cancer Ischemic heart disease Hematologic diseases (excluding hematologic malignancies) Pregnancy, childbirth, postpartum period Kidney cancer Pancreatic cancer Brain tumor Inflammatory bowel disease Liver cirrhosis Sarcoma Benign prostatic hyperplasia Ductal carcinoma in situ, benign breast tumor, breast disorders Asthma Chronic cerebrovascular disease Other liver diseases Diabetes mellitus Musculoskeletal disorders Atopic dermatitis Hepatitis (viral hepatitis) Chronic obstructive pulmonary disease (COPD) Interstitial lung disease Head and neck cancer Cerebral infarction Premature infant non-disease participants Oher diseases*	10,920 (6.27) 7490 (4.30) 7249 (4.16) 6583 (3.78) 6334 (3.64) 6046 (3.47) 5604 (3.22) 5560 (3.19) 4944 (2.84) 4576 (2.63) 4476 (2.57) 4209 (2.42) 4165 (2.39) 4132 (2.37) 3853 (2.21) 3583 (2.06) 3514 (2.02) 3382 (1.94) 2779 (1.60) 2040 (1.17) 1813 (1.04) 1684 (0.97) 1607 (0.92) 1463 (0.84) 1442 (0.83) 1340 (0.77) 1338 (0.77) 1325 (0.76) 1283 (0.74) 919 (0.53) 887 (0.51) 680 (0.39) 374 (0.21) 284 (0.16) 251 (0.14) 226 (0.13) 13 (0.01) 13,541 (7.78) 42,174 (24.23)
Total		174,083

* Top-5 diseases in other diseases included benign neoplasm of the pituitary gland (*n* = 1139), carcinoma in situ of the bladder (*n* = 965), calculus of the kidney and ureter (*n* = 796), abnormal findings on diagnostic imaging of the lungs (*n* = 721), and benign neoplasm of the adrenal gland (*n* = 513)

### Satisfaction evaluation of the KBN BRIDGE platform

A survey assessing the functionality and user satisfaction with the KBN BRIDGE system was conducted among 35 biobank institutional data managers. Among the respondents, 28 (75.7%) were female and 9 (24.3%) were male, with ages ranging between 20 and 50 years. Based on career experience, 6 respondents (17%) had less than 1 year, 12 (34%) had 1 to less than 3 years, 5 (14%) had 3 to less than 5 years, 4 (11%) had 5 to less than 10 years, and 8 (23%) had over 10 years of experience. The institutions to which the biobank institutional data manager belonged were categorized by bed capacity, with 14 respondents (40%) working in hospitals with 1,000 or more beds and 19 respondents (54%) working in hospitals with 500–999 beds (Table 4).

**Table 4:** Demographic data of the biobank institutional data manager participants in the platform’s satisfaction evaluation study

Variable	Category	Total *N* (%)
Gender	Female Male	30 (86) 5 (14)
Age (years)	21–30 31–40 41–50 Over 50	15 (43) 10 (29) 8 (23) 2 (6)
Job Career (years)	Within 1 year 1 to less than 3 years 3 to less than 5 years 5 to less than 10 years Over 10 years	6 (17) 12 (34) 5 (14) 4 (11) 8 (23)
Hospital beds	1,000 or more beds 500–999 beds 30–99 beds	14 (40) 19 (54) 2 (6)
Total		35

The average rating for the KBN BRIDGE system’s overall satisfaction was 3.59 (standard deviation [*SD*]: 0.66), based on the average scores for system satisfaction items (Table 5).

**Table 5:** Korea biobank network (KBN) biobank research information and digital image exchange (BRIDGE) platform’s satisfaction score based on a 5-point scale by item (*n* = 35)

No.	Items	Score (mean)	SD
1 2 3 4 5	I have a positive attitude towards KBN BRIDGE. I am satisfied with the efficiency of KBN BRIDGE in clinical information collection. I would like to continue using KBN BRIDGE in the future. Overall, I am satisfied with KBN BRIDGE. KBN BRIDGE allows me to use it in the way I prefer.	3.66 3.49 3.51 3.54 3.31	0.68 0.74 0.61 0.65 0.63
	Total	3.50	0.66

*SD*, standard deviation

## Discussion

Large-scale biobanks continue to face many issues in data collection, quality management, and utilization [35–38]. The KBN proposed a biobank big data platform to streamline the collection and use of extensive clinical information, and subsequently developed and evaluated the KBN BRIDGE platform.

Using KBN BRIDGE enables multiple biobanks to collect and utilize high-quality clinical information on various diseases through tasks, such as clinical data collection and management, quality assurance, and automated preprocessing for basic statistics. Specifically, the development of the KBN CDM allows for the integration of EMR and CDW data for patients with 39 diseases across 43 biobanks. As a result of our comprehensive review of various international data models for biobanks (see Additional file S1), and following consultations and consensus with multiple institutions as well as relevant governmental departments, we decided to develop a tailored model for the Korean Biobank Network. In addition, globally recognized terminologies—such as SNOMED CT, LOINC, and ATC—were applied for code mapping to provide a foundation for interoperability. This approach allows us to efficiently capture the most useful information specific to our context. Furthermore, similar to MIABIS, our model is being continuously maintained and improved to ensure long-term applicability and adaptability [39].

However, further efforts are required to achieve compatibility for international collaborative research. To this end, we are developing two complementary strategies. First, we are developing an OMOP conversion. OMOP is widely recognized as the standard for multi-institutional studies worldwide, with reported case of mapping of UK Biobank data to this model [15, 16]. While it may be challenging for the OMOP CDM to fully accommodate the structure and scope of our KBN CDM data, we are actively working on mapping to enable conversion of as much data as possible [16]. Through this conversion to OMOP CDM, we aim to strengthen international collaborative research and facilitate more effective utilization of our data. Second, we are already exploring the feasibility of applying FHIR (Fast Healthcare Interoperability Resources) for data integration. A systematic review has reported that FHIR has the potential to enhance interoperability, although most implementations remain at the prototype stage [40]. In this context, several KBN CDM tables (PERSON, SPECIMEN, DRUG, and CLINEXAM) were converted into corresponding FHIR resources, and transmissions to a FHIR server were tested. We plan to extend these efforts to a broader set of tables within the KBN CDM. Together, these efforts illustrate both the current limitations and the ongoing measures to improve compatibility with international standards.

The implementation of an ETL process allows institutions to automatically extract and transform data from their EMR and CDW, significantly enhancing data collection efficiency compared with traditional manual methods. Real-time data quality verification and the quality assurance report facilitate the identification and correction of erroneous data, emphasizing the importance of data quality management. Previously, biobank institutional data managers were required to manually review patient records and input data into spreadsheets, which was both time-consuming and labor-intensive. In contrast, the introduction of the ETL process and the quality assurance management system automates repetitive tasks, enabling the collection of a significantly larger volume of high-quality data within the same timeframe. While both approaches aim to systematically organize clinical data, the automated system has demonstrated improvements in scalability and efficiency, as evidenced by the increased data volume and enhanced quality verification. However, variations in data collection methods and infrastructure across biobanks may lead to discrepancies in data quality; this underscores the need for continuous monitoring and assessment of each biobank’s data management infrastructure and professional resources [41, 42].

The KBN BRIDGE platform shares a common objective with international biobank initiatives such as the UK Biobank, All of Us: to develop integrated infrastructures that combine large-scale clinical data and biospecimen information for research purposes. These platforms all aim to standardize and integrate various types of clinical information, including EMR or EHR data, and to provide systematic access for researchers. However, KBN BRIDGE differs in that it was designed to reflect the practical operating environments of biobanks, enabling each institution to autonomously integrate data in real time. It implements automated data collection infrastructure that includes API-based integration from hospital EMR/CDW systems, implemented dual collection methods (API and CSV) to accommodate varying institutional IT capabilities. While international platforms integrate data from hundreds of thousands of participants at the national or population level, our platform was specifically designed to integrate clinical data across 43 biobanks and 39 diseases.

In addition, KBN BRIDGE has developed a real-time data quality management system featuring automated quality validation at the point of data upload, comprehensive quality reports with visualization dashboards, and immediate error detection and correction feedback loops. Building on these capabilities, KBN BRIDGE has established a comprehensive infrastructure that supports scalable and sustainable clinical data management across the national biobank network. Currently, the platform processes data from 136,473 patients and manages over 1.4 million biospecimens, demonstrating its capacity to handle large-scale data integration. Furthermore, it includes automated statistical preprocessing functions, enabling users to generate summary statistics and explore dataset characteristics without the need for programming skills. This transformation has shifted the previously reported manual and fragmented data collection system by Ko et al. (2021) into an automated and scalable big data platform [20]. By doing so, KBN BRIDGE not only enhances data accessibility and usability, but also strengthens the foundation for collaborative studies and data-driven precision medicine efforts in Korea.

The KBN BRIDGE platform facilitates the integration of clinical information collected by biobanks across Korea, yet currently restricts each institution to accessing its own data only. Biobanks typically limit data access to protect donor-provided data, preventing misuse and ensuring data security. Given the importance of establishing trust with donors, biobanks often provide data only to approved researchers for specific research purposes [43].

To address the platform’s limitations and enhance research collaboration, it is necessary to consider an open-access approach that allows data to be used by researchers across multiple institutions rather than being restricted to specific institutional researchers. For example, the UK Biobank has implemented an open-access system that enables researchers worldwide to access its data, facilitating greater research and collaboration [44]. Additionally, it has developed a research analysis platform that allows researchers to analyze and collaborate on data within a cloud environment [45]. Such examples serve as important reference models for expanding data usability by implementing a research analysis platform within KBN BRIDGE; this would enable approved KBN researchers to search and analyze data for approved research purposes, share data, and collaborate on joint research projects. Nevertheless, to reduce data usage barriers, the KBN BRIDGE platform allows users to download basic statistical preprocessing files, enabling them to examine the distribution of desired datasets and statistics. This demonstrates the potential for data collected by the KBN BRIDGE to be utilized across various research fields and disease studies.

Another key finding of this study is the overall satisfaction assessment of institution data managers. The average satisfaction score was 3.50, positioned between *neutral (3)* and *agree (4)* on a 5-point Likert scale. The highest score was observed for the item “I have a positive attitude towards KBN BRIDGE” (3.66), while the lowest score was for “KBN BRIDGE allows me to use it in the way I prefer” (3.31). This contrast suggests a potential need for improvement in terms of user interface or functional flexibility, even as users acknowledged the value and direction of the platform overall. The 35 survey participants were data managers employed at a biobank, primarily tasked with operational duties rather than research activities.

Prior to the implementation of KBN BRIDGE, institutions manually extracted clinical data from electronic medical record systems and organized it using spreadsheet-based methods. With the transition to an automated platform, users needed to adapt to new workflows, which included understanding CDM mapping logic, interpreting automatically generated reports, navigating API-based protocols, and managing standardized coding systems. We conducted training sessions throughout this process and gathered feedback from stakeholders; however, we faced challenges due to the technical complexity involved. In particular, balancing the requirements for data standardization with the flexibility of the institutions emerged as a significant conflict. The observed satisfaction scores reflected the gap in users’ technical capabilities and readiness, a common challenge in the digital transformation of biobank systems. These results indicate that ongoing user engagement and interest are crucial for the successful implementation of such platforms.

Due to the limited sample size of users in this study, the satisfaction results may not fully represent the experiences of the entire user base. Therefore, it is necessary to secure a larger and more representative sample to evaluate satisfaction and analyze the factors that affect system satisfaction, which can help identify areas for improvement. Various studies are being conducted to overcome the limitations of generalizability in research with limited user samples, which is also an important consideration in evaluating specific biobanks. The KBN BRIDGE platform shows potential as a valuable system for users, although further studies are needed to validate its effectiveness and user satisfaction.

## Conclusions

The KBN BRIDGE platform provides an efficient system for collecting, managing, and utilizing large-scale clinical information on 39 diseases across 43 biobanks within the KBN, thereby laying a foundation for positive biobank research through practical application. By introducing an automated approach, the platform streamlined data processes by supporting the integration and standardization of clinical information while offering a unified environment equipped with quality management and analysis functions. These advancements enable researchers to access comprehensive datasets with minimal effort and perform basic analyses without requiring complex technical skills. Future efforts to refine the KBN BRIDGE platform should focus on system improvements and advancing data-sharing frameworks, enabling the platform to play an increasingly vital role in Korean biobank research.

## Supplementary Information

Below is the link to the electronic supplementary material.

Supplementary Material 1

## Data Availability

The datasets used and/or analyzed during the current study are not publicly available as they are obtained directly from participating institutions within the platform (https://www.kbn-clindb.re.kr/kbn/). However, they may be available from the corresponding author on reasonable request, subject to the approval of the respective institutions and compliance with data sharing agreements.

## Abbreviations

HTML5: Hypertext markup language 5
SSL: Secure sockets layer
VPN: Virtual private network
OTP: One-time password
API: Application programming interface
BRIDGE: Biobank Research Information and Digital Image Exchange platform
CDM: Common data model
CDW: Clinical data warehouse
CSV: Comma-separated value
EHR: Electronic health record
EMR: Electronic medical record
ETL: Extract, transform, and load
KBN: Korea Biobank Network
HuBIS_Sam: Human Biobank Information System for Sample
OMOP: Observational medical outcomes partnership
i2b2: Informatics for integrating biology and the bedside
MIABIS: Minimal information about biobank data sharing
SNOMED CT: Systematized nomenclature of medicine clinical terms
LOINC: Logical observation identifiers names and codes
ATC: Anatomical therapeutic chemical classification system
FHIR: Fast Healthcare Interoperability Resources

## References

[1] Coppola L, et al. Biobanking in health care: evolution and future directions. J Transl Med. 2019;17(1):172. 31118074 10.1186/s12967-019-1922-3 PMC6532145

[2] Paskal W, et al. Aspects of modern biobank Activity - Comprehensive review. Pathol Oncol Res. 2018;24(4):771–85. 29728978 10.1007/s12253-018-0418-4 PMC6132819

[3] Malsagova K et al. Biobanks-A platform for scientific and biomedical research. Diagnostics (Basel). 2020;10(7). 10.3390/diagnostics10070485 PMC7400532 32708805

[4] Beesley LJ, et al. The emerging landscape of health research based on biobanks linked to electronic health records: existing resources, statistical challenges, and potential opportunities. Stat Med. 2020;39(6):773–800. 31859414 10.1002/sim.8445 PMC7983809

[5] Eminaga O, et al. Linkage of data from diverse data sources (LDS): a data combination model provides clinical data of corresponding specimens in biobanking information system. J Med Syst. 2013;37(5):9975. 24022214 10.1007/s10916-013-9975-y

[6] Späth MB, Grimson J. Applying the archetype approach to the database of a biobank information management system. Int J Med Inf. 2011;80(3):205–26. 10.1016/j.ijmedinf.2010.11.002 21131230

[7] Lähteenmäki J, et al. Integrating data from multiple Finnish biobanks and National health-care registers for retrospective studies: practical experiences. Scand J Public Health. 2022;50(4):482–9. 33845693 10.1177/14034948211004421 PMC9152591

[8] Lewis C, et al. Building a ‘repository of science’: the importance of integrating biobanks within molecular pathology programmes. Eur J Cancer. 2016;67:191–9. 27677055 10.1016/j.ejca.2016.08.009

[9] Kinkorova J, Topolcan O. Biobanks in the era of big data: objectives, challenges, perspectives, and innovations for predictive, preventive, and personalised medicine. EPMA J. 2020;11(3):333–41. 32849924 10.1007/s13167-020-00213-2 PMC7429593

[10] Chua SYL, et al. Cohort profile: design and methods in the eye and vision consortium of UK biobank. BMJ Open. 2019;9(2):e025077. 30796124 10.1136/bmjopen-2018-025077 PMC6398663

[11] Darke P, et al. Curating a longitudinal research resource using linked primary care EHR data-a UK biobank case study. J Am Med Inf Assoc. 2022;29(3):546–52. 10.1093/jamia/ocab260 PMC8800530 34897458

[12] Nagai A, et al. Overview of the biobank Japan project: study design and profile. J Epidemiol. 2017;27(3S):S2–8. 28189464 10.1016/j.je.2016.12.005 PMC5350590

[13] Klann JG, et al. Data model harmonization for the all of Us research program: transforming i2b2 data into the OMOP common data model. PLoS ONE. 2019;14(2):e0212463. 30779778 10.1371/journal.pone.0212463 PMC6380544

[14] Mayo KR, et al. The all of Us data and research center: creating a secure, scalable, and sustainable ecosystem for biomedical research. Annu Rev Biomed Data Sci. 2023;6:443–64. 37561600 10.1146/annurev-biodatasci-122120-104825 PMC11157478

[15] OHDSI. The book of OHDSI. Observational Health Data Sciences and Informatics; 2019.

[16] Michael CL et al. Mapping local biospecimen records to the OMOP common data model. AMIA Jt Summits Transl Sci Proc. 2020:422. PMC7233045 32477663

[17] Murphy SN, et al. Serving the enterprise and beyond with informatics for integrating biology and the bedside (i2b2). J Am Med Inf Assoc. 2010;17(2):124–30. 10.1136/jamia.2009.000893 PMC3000779 20190053

[18] Norlin L, et al. A minimum data set for sharing biobank samples, information, and data: MIABIS. Biopreserv Biobank. 2012;10(4):343–8. 24849882 10.1089/bio.2012.0003

[19] Cho SY, et al. Opening of the National biobank of Korea as the infrastructure of future biomedical science in Korea. Osong Public Health Res Perspect. 2012;3(3):177–84. 24159511 10.1016/j.phrp.2012.07.004 PMC3738708

[20] Ko S-J et al. Common data model and database system development for the Korea biobank network. Appl Sci. 2021;11(24).

[21] Chatterjee A, Prinz A. Applying Spring Security framework with Keycloak-based OAuth2 to protect microservice architecture APIs: a case study. Sensors (Basel). 2022;22(5):1883.. 10.3390/s22051703 PMC8914669 35270850

[22] Reisinger SJ, et al. Development and evaluation of a common data model enabling active drug safety surveillance using disparate healthcare databases. J Am Med Inf Assoc. 2010;17(6):652–62. 10.1136/jamia.2009.002477 PMC3000752 20962127

[23] Eklund N, et al. Extending the minimum information about biobank data sharing terminology to describe samples, sample donors, and events. Biopreserv Biobank. 2020;18(3):155–64. 32302498 10.1089/bio.2019.0129 PMC7310316

[24] Vuokko R, et al. Systematized nomenclature of Medicine–Clinical terminology (SNOMED CT) clinical use cases in the context of electronic health record systems: systematic literature review. JMIR Med Inf. 2023;11:e43750. 10.2196/43750 PMC9941898 36745498

[25] Bakken S, et al. Evaluation of the clinical LOINC (Logical observation identifiers, names, and Codes) semantic structure as a terminology model for standardized assessment measures. J Am Med Inf Assoc. 2000;7(6):529–38. 10.1136/jamia.2000.0070529 PMC129661 11062226

[26] Zhu Q et al. Standardized drug and pharmacological class network construction. MEDINFO. 2013:1125–1129. PMC3909176 23920899

[27] Kim JO, et al. Professional biobanking education in Korea based on ISO 20387. J Pathol Transl Med. 2025;59(1):11–25. 39815742 10.4132/jptm.2024.11.04 PMC11736279

[28] Kaspar M, et al. Automated provision of clinical routine data for a complex clinical follow-up study: A data warehouse solution. Health Inf J. 2022;28(1):14604582211058081. 10.1177/14604582211058081 34986681

[29] Lynch KE, et al. Incrementally transforming electronic medical records into the observational medical outcomes partnership common data model: A multidimensional quality assurance approach. Appl Clin Inf. 2019;10(5):794–803. 10.1055/s-0039-1697598 PMC6811349 31645076

[30] Kim KH, et al. Healthcare data quality assessment for improving the quality of the Korea biobank network. PLoS ONE. 2023;18(11):e0294554. 37983215 10.1371/journal.pone.0294554 PMC10659164

[31] Garcia-Smith D, Effken JA. Development and initial evaluation of the clinical information systems success model (CISSM). Int J Med Inf. 2013;82(6):539–52. 10.1016/j.ijmedinf.2013.01.011 23497819

[32] Kuo KM et al. Strategic improvement for quality and satisfaction of hospital information systems. J Healthc Eng. 2018:3689618. 10.1155/2018/3689618 PMC6157169 30298099

[33] He K, et al. Decoding the glycoproteome: a new frontier for biomarker discovery in cancer. J Hematol Oncol. 2024;17(1):12. 38515194 10.1186/s13045-024-01532-x PMC10958865

[34] Hong J, et al. Multiomics profiling of Buffy coat and plasma unveils etiology-specific signatures in hepatocellular carcinoma. Clin Mol Hepatol. 2024;30(3):360–74. 38486508 10.3350/cmh.2024.0042 PMC11261225

[35] Annaratone L, et al. Basic principles of biobanking: from biological samples to precision medicine for patients. Virchows Arch. 2021;479(2):233–46. 34255145 10.1007/s00428-021-03151-0 PMC8275637

[36] Muller H, et al. Biobanks for life sciences and personalized medicine: importance of standardization, biosafety, biosecurity, and data management. Curr Opin Biotechnol. 2020;65:45–51. 31896493 10.1016/j.copbio.2019.12.004

[37] Rush A, et al. Improving academic biobank value and sustainability through an outputs focus. Value Health. 2020;23(8):1072–8. 32828220 10.1016/j.jval.2020.05.010

[38] Schuttler C, et al. The journey to Establishing an IT-infrastructure within the German biobank alliance. PLoS ONE. 2021;16(9):e0257632. 34551019 10.1371/journal.pone.0257632 PMC8457464

[39] Eklund N, et al. Update of the minimum information about biobank data sharing (MIABIS) core terminology to the 3rd version. Biopreserv Biobank. 2024;22(4):346–62. 38497765 10.1089/bio.2023.0074

[40] Vorisek C, et al. Fast healthcare interoperability resources (FHIR) for interoperability in health research: systematic review. JMIR Med Inf. 2022;10(7):e35724. 10.2196/35724 PMC9346559 35852842

[41] Characterizing biobank organizations in the U.S._results from a national survey.pdf. 10.1186/gm407 PMC3706795 23351549

[42] Li H, et al. A survey of the current situation of clinical biobanks in China. Biopreserv Biobank. 2017;15(3):248–52. 28080144 10.1089/bio.2016.0095

[43] Milne R, Sorbie A, Dixon-Woods M. What can data trusts for health research learn from participatory governance in biobanks? J Med Ethics. 2022;48(5):323–8. 33741681 10.1136/medethics-2020-107020 PMC9046739

[44] Conroy M, et al. The advantages of UK biobank’s open-access strategy for health research. J Intern Med. 2019;286(4):389–97. 31283063 10.1111/joim.12955 PMC6790705

[45] Conroy MC, et al. UK biobank: a globally important resource for cancer research. Br J Cancer. 2023;128(4):519–27. 36402876 10.1038/s41416-022-02053-5 PMC9938115

