# Supplementary material for: Development of a big data platform for collecting and utilizing clinical information from the Korea Biobank Network
Source: BMC Med Inform Decis Mak. 2025 Oct 8;25:366. doi: 10.1186/s12911-025-03192-4 (PMC12505599; doi:10.1186/s12911-025-03192-4)
Supplement: Supplementary file 1 — Supplementary Material 1 [file 12911_2025_3192_MOESM1_ESM.docx]

**Table S1: Comparison of existing international data models and the KBN Common Data Model.**

This table compares representative international data models relevant to biobank research, including the Observational Medical Outcomes Partnership (OMOP), Informatics for Integrating Biology and the Bedside (i2b2), Minimum Information About Biobank Data Sharing (MIABIS) 2012, MIABIS 2020 extensions, and the Korean Biobank Network (KBN) Common Data Model(CDM) developed in this study.

| **Model** | **Primary scope** | **Data granularity** | **Biospecimen coverage** | **Clinical coverage examples** | **Specimen–clinical linkage** | **Representative use examples** |
| --- | --- | --- | --- | --- | --- | --- |
| **OMOP CDM (v5.3)** | Clinical analytics of EHR/claims | Individual-level | Specimen domain present; limited adoption in biobank context | Rich: diagnoses, drugs, procedures, labs, observations, etc | Yes (via person_id) | Multi-site observational studies (OHDSI network) |
| **i2b2** | Cohort discovery (local & federated) | Individual-level (site ETL) | No standardized biospecimen domain; biobank portals use custom LIMS → i2b2 | Moderate: depends on local ETL/ontology | Limited; site-specific (no standard schema) | Institutional cohort queries |
| **MIABIS 2012** | Biobank registry/catalog | Collection-level (aggregate) | Yes: collection-level attributes (sample type, storage, diagnosis) | None (no individual clinical data) | None (no patient linkage) | BBMRI-ERIC Directory, national biobank catalogs |
| **MIABIS 2020 (Extensions)** | Biobank metadata with individual-level query | Individual-level (Sample Donor / Sample / Event) | Yes: detailed type, storage temperature, anatomical site (SPREC-based) | Limited: donor demographics, diagnosis, events  (not full EHR) | Yes (donor–sample–event structure) | BBMRI-ERIC Sample Locator platform |
| **KBN CDM (this study)** | Biobank–clinical integration with EMR/CDW (Korea) | Individual-level | Yes: *HuBIS_Sam–linked specimen codes, disease-specific follow-up | Broad: 38 diseases, labs, drugs, history, notes, follow-up, etc | Yes (PERSON–REGISTRATION–SPECIMEN) | Multi-institution korean biobank integration (43 biobanks) |

* Human Biobank Information System for Sample (HuBIS_Sam): a human biospecimen management program used by the Korea National Biobank to collect, store, manage quality, and distribute biospecimens

- The OMOP is the standard model developed by Observational Health Data Sciences and Informatics (OHDSI) to harmonize observational health data for large-scale clinical analytics. It captures individual-level data across multiple domains, including conditions, drugs, procedures, labs, and observations [1]. While a dedicated Specimen domain exists and links specimens to clinical records via person_id, adoption for biobank operations has been limited [2].
- The i2b2 is primarily designed to support cohort discovery at both local and federated levels. It stores individual-level patient data, but the coverage of domains depends on each site’s Extract–Transform–Load (ETL) and ontology mapping. However, there is no standardized support for biospecimen data, which restricts its direct use in biobank operations [3].
- The MIABIS 2012 is provides a minimal information standard for describing biobanks and sample collections, with 52 attributes designed for registry- and catalog-level descriptions. It includes sample types, storage, and diagnoses at the collection level but does not contain individual-level clinical or specimen linkages. It has been widely applied in Biobanking and BioMolecular resources Research Infrastructure – European Research Infrastructure Consortium (BBMRI-ERIC) directories and other biobank registries [4].
- The MIABIS 2020 introduced the Sample Donor, Sample, and Event components, enabling individual-level representation of donors, biospecimens, and associated events (e.g., diagnoses, collection, death). The model incorporates Standard PREanalytical Code (SPREC)-based attributes such as sample type, storage temperature, and anatomical site. Clinical attributes remain minimal, and the framework explicitly recommends mapping MIABIS to clinical data model such as Fast Healthcare Interoperability Resources (FHIR) and open Electronic Health Records (open EHR) to ensure interoperability [5].
- The KBN CDM, as presented in this study, was designed to meet the operational requirements of the KBN. It integrates biospecimen and clinical information at the individual level through PERSON–REGISTRATION–SPECIMEN structures, linking Human Biobank Information System for Sample (HuBIS_Sam)-managed specimen codes with rich clinical data across 38 diseases and non-disease participants.

**References**

1. OHDSI, The Book of OHDSI. Observational Health Data Sciences and Informatics, 2019.

2. Michael, C.L., et al., *Mapping local biospecimen records to the OMOP common data model.* AMIA Jt Summits Transl Sci Proc, 2020: p. 422.

3. Murphy, S.N., et al., *Serving the enterprise and beyond with informatics for integrating biology and the bedside (i2b2).* J Am Med Inform Assoc, 2010. **17**(2): p. 124-130.

4. Norlin, L., et al., *A minimum data set for sharing biobank samples, information, and data: MIABIS.* Biopreserv Biobank, 2012. **10**(4): p. 343-348.

5. Eklund, N., et al., *Extending the Minimum Information About BIobank Data Sharing Terminology to Describe Samples, Sample Donors, and Events. Biopreserv Biobank*, 2020. **18**(3): p. 155–164.
